# Synthesis and Dissolution of Metal Oxides in Ionic Liquids and Deep Eutectic Solvents

**DOI:** 10.3390/molecules25010078

**Published:** 2019-12-24

**Authors:** Janine Richter, Michael Ruck

**Affiliations:** 1 Faculty of Chemistry and Food Chemistry, Technische Universität Dresden, 01062 Dresden, Germany; janine.richter4@tu-dresden.de; 2 Max Planck Institute for Chemical Physics of Solids, Nöthnitzer Strasse 40, 01187 Dresden, Germany

**Keywords:** ionic liquid, deep eutectic solvent, metal oxide, dissolution

## Abstract

Ionic liquids (ILs) and deep eutectic solvents (DESs) have proven to be suitable solvents and reactants for low-temperature reactions. To date, several attempts were made to apply this promising class of materials to metal oxide chemistry, which, conventionally, is performed at high temperatures. This review gives an overview about the scientific approaches of the synthesis as well as the dissolution of metal oxides in ILs and DESs. A wide range of metal oxides along with numerous ILs and DESs are covered by this research. With ILs and DESs being involved, many metal oxide phases as well as different particle morphologies were obtained by means of relatively simple reactions paths. By the development of acidic task-specific ILs and DESs, even difficultly soluble metal oxides were dissolved and, hence, made accessible for downstream chemistry. Especially the role of ILs in these reactions is in the focus of discussion.

## 1. Introduction

Metal oxide materials are of great scientific as well as economic interest because of their versatile utilisation and potential new applications. They are used as functional materials as well as starting substances for the synthesis of diverse task-specific materials. Thereby, their phase, morphology, particle size and purity, often, are of significant importance. Especially for applications, such as catalysis, photovoltaics, and batteries, it is essential to obtain metal oxide particles with specific characteristics, which give control over optical and electronic properties [1]. Thus, for example ZnO, a promising material in the fields of optoelectronics because of its wide band gap (3.3 eV) [2], was synthesised in nano-sized wire [3], rod [4], and tetrapod [5] morphology via template-based sol-gel methods [3], polymer-assisted solution-phase routes [4] and chemical vapour transport [5], respectively. Block-copolymers as a structure-directing template for mesostructured materials have already previously been applied to the synthesis of a large number of metal oxides including Al_2_O_3_, HfO_2_, Nb_2_O_5_, SnO_2_, Ta_2_O_5_, TiO_2_, WO_3_ and ZrO_2_, as well as the mixed oxides Al_2_TiO_x_, SiAlO_x_, SiTiO_x_, ZrTiO_x_ and ZrW_2_O_x_ [6]. Furthermore, rare earths, such as CeO_2_, are of increasing interest for a wide range of applications, e.g., as catalysts, oxygen sensors, and UV absorbers [7]. To synthesise these materials in desired morphologies, hydrothermal [8] and solvothermal [9] methods, microwave-assisted hydrothermal methods [10], thermal decomposition of precursors [11], the sol-gel process [12] and gas condensation [13] have been used. However, these methods often have some drawbacks like high temperatures or pressures, toxic and volatile organic solvents, and expensive starting materials.

Besides their (potential) industrial applications, metal oxides are also important starting materials for the synthesis of metals or diverse metal compounds. Based on naturally occurring ores, earths and minerals, the production of these chemicals, usually, involves high temperatures. Industrially very important examples are iron, aluminium and titanium [14] which are produced from their oxidic ores at temperatures around 1000 °C [15]. With fossil fuels still dominating the energy market, the large amounts of energy consumed involves high CO_2_ emissions. Furthermore, hydrometallurgical methods are applied [16], however, this often is accompanied by the simultaneous dissolution of undesired materials [17]. In addition, metal oxides are present in nuclear waste, where large amounts of secondary waste are generated in the course of metal extraction [18].

In times of climate change and an increasing shortage of non-renewable energy resources as well as waste problems in many parts of the world, the high energy and resource demand for the synthesis as well as the processing of metal oxides has to be reconsidered. Promising alternative solvents for inorganic reactions at low temperatures are ionic liquids (ILs) and deep eutectic solvents (DESs). ILs, by definition, are salts with a melting point below 100 °C. Their favourable properties, such as a negligible vapour pressure, a wide liquid range, good thermal and chemical stability as well as the ability to dissolve a large number of substances, make them an interesting reaction medium for low-temperature synthesis [19]. Similar properties are found for DESs, which are eutectic mixtures of Brønsted or Lewis acids and bases that contain large, asymmetric ionic species. The crucial difference between ILs and DESs is the wide variety of ionic species present in DESs, while ILs mainly consist of one discrete type of anion and cation [20]. 

**Figure 1:**
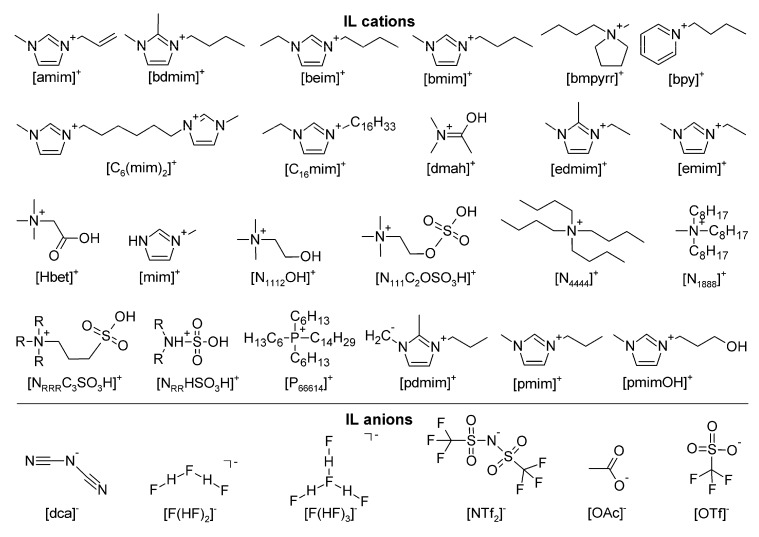
Structures of IL cations and anions addressed in this review.

The present review article summarises the current state of knowledge on metal oxide chemistry in ILs and DESs in order to provide researchers an overview about the synthetic approaches. Several review articles cover some fields in metal oxide chemistry [21,22,23], however, no comprehensive summary about the state of knowledge regarding this topic exists. In the following, the first section is concerned with the synthesis of metal oxides, while the second part introduces the dissolution chemistry of metal oxides in ILs and DESs. An overview about the abbreviations used in this article is given in Table 1. The structural formulas of cations and anions of the discussed ILs are presented in Figure 1. The focus is on metal oxide chemistry, therefore, metalloids mostly are neglected.

## 2. Synthesis of Metal Oxides

### 2.1. Reactions in ILs

To date, several methods have been published with ILs being used to synthesise metal oxides. However, often ILs merely act as a template to obtain desired phases or morphologies of metal oxides and are not essential for the reaction itself. Especially a better control of nanoparticle synthesis by ILs has been of great scientific interest. Because of the formation of extended hydrogen bond systems in other solvents, many ILs are suitable templates, providing a highly structured reaction environment [24]. Especially in solvothermal synthesis, ILs are added in order to form suitable templates [25]. However, this review is not concerned with the template effect of small amounts of ILs as additives, therefore, in the following, we will not consider reactions in solutions of water or other solvents as major component. Instead, the focus will be on the synthesis of metal oxides in ILs acting as solvent or even reaction partner.

Three main synthesis methods in ILs are popular, which are ionothermal, microwave-assisted and sonochemical reactions. Based on the hydrothermal reactions, ionothermal means the heating of a reaction mixture in an IL instead of water as a reaction medium. Because of the low vapour pressure of ILs, no high autogenous pressure builds up, which is why ionothermal reactions often take place at or close to ambient pressure [26]. ILs are particularly suitable for microwave heating. Because of their large, highly polarisable cations, a very efficient absorption of microwave irradiation makes them an effective heating medium for microwave-assisted synthesis [27]. Recently, also the combination of sonochemistry and ILs has been reported [28,29,30,31]. Based on the phenomenon of acoustic cavitation, ultrasound irradiation produces so-called cavitation bubbles which generate very high temperatures and pressures when collapsing [32,33]. All three methods are applied to metal oxide synthesis in ILs.

#### 2.1.1. Titanium Oxide

Among the metal oxides synthesised in ILs, TiO_2_ plays a major role, as already highlighted in a review by Voepel and Smarsly [34]. TiO_2_ exists in several phases, the most common ones being rutile, anatase and brookite. In aqueous medium, rutile is the most stable phase for macroscopic particles. However, decreasing the size to nanoparticles, anatase is favoured [35].

The general procedure for the IL-assisted synthesis of TiO_2_ is the dissolution of either TiCl_4_ or Ti(O^i^Pr)_4_ in a mixture of an IL and water and subsequent heating. It is apparent from the fact that water alone hydrolyses these titanium salts very readily to TiO_2_ [36], that ILs in these systems merely act as templates. Nevertheless, a wide variety of morphologies as well as improved phase control is accessible by IL-assisted syntheses. 

Using ionothermal heating of TiCl_4_ (100 °C, 14 h), Smarsly et al. found, that the phase of titania nanoparticles is controllable by the IL’s anion. Apparently, the [NTf_2_]^−^ anions of the ILs [emim][NTf_2_], [bmpyrr][NTf_2_] and [P_66614_][NTf_2_] stabilise an amorphous TiO_2_ phase which spontaneously crystallises into rutile during IL extraction with isopropyl alcohol. The [NTf_2_]^−^ anion could either be an acceptor for the protons forming during the reaction, subsequently, acting as a surfactant, or directly interact with the titania species. Comparing the (110) surface of rutile and anatase, a significantly higher amount of unsaturated titanium atoms is found for the former, which readily react with the electron lone pair of [NTf_2_]^−^. Thus, assumedly, in the presence of [NTf_2_]^−^, an intermediate amorphous titanium oxide phase is formed, which favours the subsequent crystallisation into rutile. For this reaction to take place, also the amount of water has to be kept low, in order to avoid the formation of anatase particles usually taking place in aqueous medium. In contrast to that, reactions in [bmim][BF_4_], [C_16_mim]Cl and [emim][BF_4_] yield anatase [37]. 

The same result was found by Zhou and Antonietti, using TiCl_4_ and the IL [bmim][BF_4_] as the starting materials and an ionothermal approach (80 °C, 12 h) to obtain anatase nanoparticles that assemble to mesoporous spherical particles. The IL, apparently, favours a reaction-limited aggregation mechanism, resulting in very small primary particles with a size of only 2–3 mm [38]. 

Using the same IL, Liu et al. synthesised phase-pure anatase nanoparticles from Ti(O^i^Pr)_4_ in a microwave-assisted heating method (126 W, 40 min). According to them, the hydrolysis of Ti(O^i^Pr)_4_ leads to the condensation into short-chain polyanions, which are protected as dissolved intermediates by [bmim]^+^. Only with a fast temperature increase, rapid crystal growth occurred. In this process, the [bmim]^+^ cation, apparently, protects the seed crystals, as less preferred crystal growth was observed, yielding truncated bipyramid particles [39]. 

Furthermore, anatase was synthesised by Mudring et al. in a sonochemical reaction (45 kHz, 60 W, 9 h) by dissolving Ti(O^i^Pr)_4_ in the IL [pmimOH][NTf_2_]. With a particle size of 5 nm, anatase is the most stable product phase to be expected, however, the synthesis at ambient conditions poses some difficulties, that can be overcome by this IL-assisted, sonochemical method. Furthermore, the IL as a template supports the formation of spherical particles [31]. The [pmimOH]^+^ cation owns not only an acidic proton at the imidazolium core, but also a hydroxyl group, that could offer strong interactions [40].

Additionally, the group presented a systematic study about the synthesis of TiO_2_ in ILs and the influence of the reaction parameters, such as the titanium precursor, the cation of the IL, the heating method and the pH value. The different ILs were chosen in order to provide different ways of potential interaction: via π-π-interactions of an aromatic core ([bpy][NTf_2_]), an aromatic system and an acidic proton ([bmim][NTf_2_]), none of both ([N_1888_][NTf_2_], [P_66614_][NTf_2_]) or an additional functional group ([pmimOH][NTf_2_]). Apparently, by sonochemical heating (45 kHz, 60 °C, RT, 9 h) of Ti(O^i^Pr)_4_ in every IL, different product morphologies were obtained, highlighting the different interactions between the IL cation the product particles. Furthermore, only [pmimOH] supports the formation of phase pure anatase, while in all other ILs, mixtures of brookite and anatase are formed. The use of TiCl_4_ as starting material, instead, favours the formation of rutile. This is attributed to the formation of HCl during hydrolysis. In agreement with this, pH investigations yielded rutile by the addition of HCl, but anatase when adding urea. Interestingly, this is contrary to the previously mentioned reports [37,38], where anatase was obtained despite using a chloride IL or TiCl_4_ as the starting material. Investigating the heating method, sonochemical and ionothermal (170 °C, 2 h) heating gave anatase particles varying morphologies, however, a microwave-heated (2.45 GHz, 80 °C, 10 min) sample contained significant amounts of brookite alongside [40]. This extensive study shows that the control of phase and morphology of metal oxides in ILs depends on various factors. General statements are difficult to make as the underlying mechanisms for the effects of reaction partners und conditions are not understood, yet. It might be an interesting observation, that [pmimOH][NTf_2_], strongly interacting because of its functional group, shows the most structure-directing effect, yielding no side phases. In contrast to the report by Smarsly et al. [37], the [NTf_2_]^−^ anions of the ILs used did not support the crystallisation in rutile phase. Apparently, the higher reaction temperature or other heating methods suppress the structure-directing effect of this anion.

A different approach, without heating even being necessary, was reported by Nakashima and Kimizuka who used an interfacial sol-gel process to synthesise hollow TiO_2_ microspheres. For this purpose, they added a solution of Ti(OBu)_4_ in toluene to the IL [bmim][PF_6_] under constant stirring. As toluene is poorly soluble in the IL, a microemulsion formed with Ti(OBu)_4_ being hydrolysed by water traces at the tolouene droplet-[bmim][PF_6_] interface. The obtained gel consists of amorphous TiO_2_ and can be transformed into anatase by calcination (500 °C, 5 h). According to the authors, this method can also be applied to Hf(OBu)_4_, InSn_3_(OR)_x_, Nb(OBu)_5_ and Zr(OBu)_4_ [41].

Another interesting field in TiO_2_ chemistry is the synthesis of the metastable bronze phase TiO_2_(B) because of its lithium ion storage capacity [42,43,44] and potential photocatalytic applications [45,46]. Several research groups have synthesised this compound with a similar ionothermal method by adding TiCl_4_ and a small amount of water for hydrolysis to a mixture of [bmim]Cl, [bmim][BF_4_], [C_16_mim]Cl and [C_16_mim][BF_4_] at 95 °C [46,47,48,49,50]. Smarsly et al. identified the partly hydrolysis of the [BF_4_]^−^ anion and the subsequent provision of fluoride ions as crucial for the formation of TiO_2_(B). According to them, fluoride through coordination with titanium inhibits the formation of titanium oxyhydroxy clusters supporting the formation of the layered TiO_2_(B) structure. Depending in the ratio of the four ILs, a different product composition ranging from phase pure TiO_2_(B) to anatase, rutile or titanium hydroxyloxyfluoride as additional phases, is found [48]. This research highlights additional difficulties for the prediction of reaction outcomes when working in mixtures of different ILs as they might show a non-linear behaviour upon mixing in varying compositions. Due to the complexity of this system, further investigations need to reveal the reaction mechanisms present.

#### 2.1.2. Zinc Oxide

Mesocrystals of ZnO were synthesised in the highly hydrated IL [N_4444_]OH (often abbreviated as TBAH), as shown by Taubert et al. Refluxing a solution of Zn(CH_3_COO)_2_ in [N_4444_]OH (100 °C, 20 h) yielded nano-sized ZnO particles. For low Zn(CH_3_COO)_2_ concentrations, these primary particles assembled to hollow spheres on the glass wall of the reaction vessel. A large effect on the product morphology is ascribed to the intrinsic electric fields of the polar ZnO lattice. Thereby, the [N_4444_]^+^ cation might reverse the polarity of the individual particles, thus, preventing further growth and supporting the aggregation to a mesostructure [51]. The product morphology strongly depends on the reaction temperature, as after three days at room temperature, the rod-like primary particles assembled to solid mesocrystals. [52]. Previously, the group had shown that ZnO can also emerge from polymer-controlled reactions of aqueous Zn(NO_3_)_2_ solutions that are heated to 90 °C [53,54,55], but mesocrystals like the ones observed require the presence of [N_4444_]OH [52]. 

A sonochemical synthesis (45 kHz, 60 W, RT, 12/24 h) for ZnO was found by Mudring et al. Starting from the acetate salt, phase-pure wurtzite nanorods were obtained in the IL [bmim][NTf_2_] in the presence of NaOH [28]. Presumably, the IL in this case merely acts as a solvent and the reaction forming the metal oxide might be ascribed to the high temperatures occurring because of the ultrasound irradiation and leading to the decomposition of the acetates. In experiments, temperatures of several thousand Kelvin in the cavitation bubbles were found [32], while for Zn(CH_3_COO)_2_ a complete decomposition to ZnO was reported at 530 °C [56]. 

ZnO was also synthesised by Zheng et al. in the ILs [emim][BF_4_], [bmim][BF_4_] and [bdmim][BF_4_] from Zn(CH_3_COO)_2_·2H_2_O (80 °C, 48 h) [57]. With the underlying synthesis method, a low-temperature solid state reaction, nanocrystals of metal oxides can be produced from hydrated metal salts in the presence on NaOH [58]. However, the presence of ILs provides suitable templates for the synthesis of tailored 1d nanostructures. On one side, the presence of a proton at the C2 position of the imidazolium cation is crucial for the ability to form hydrogen bonds to the oxygen atoms of ZnO, thus, influencing the growing direction of the particle. On the other side, also the length of the alkyl chain on the imidazolium ring can affect the particle size because of steric hindrance [57].

In another attempt to synthesise ZnO, Dai et al. investigated ILs not only acting as a solvent but as a precursor for the product. The synthesised tailored ILs consist of complex zinc-alkylamine cations and [NTf_2_]^−^ anions. Together with a small amount of tetramethylammonium hydroxide, enabling nucleation formation through hydrolysis, the IL yields ZnO under ionothermal conditions (110 °C, several hours). The products are micrometer-sized particles consisting of nanostructures. The morphology strongly depends on various factors, including ligand size and temperature as the most affecting [59]. Using such approaches with the metal cation being part of the IL, the synthetic effort has to be considered as well as the fact that the reaction takes places under consumption of the IL. Regarding the previously mentioned studies, more efficient synthesis methods are assumed to be developable.

#### 2.1.3. Copper Oxide

Two synthetically easy, low temperature-approaches similar to the methods for ZnO formation were applied to CuO as well. Hence, CuO can be synthesised in the IL [N_4444_]OH ionothermally from Cu(CH_3_COO)_2_. Mechanistic studies revealed the completion of the reaction to CuO already after one minute at 40 °C and even faster at higher temperatures. The formation of the product takes place in a two-step reaction with intermediate Cu(OH)_2_ rods which subsequently aggregate and dehydrate to single-crystalline CuO nanoplates. However, a subsequent heat treatment at 250 °C to 300 °C is necessary in order to remove adherent traces of the IL [60].

Mudring et al. also synthesised CuO sonochemically from Cu(CH_3_COO)_2_ in the presence of NaOH (45 kHz, 60 W, RT, 12/24 h). While the decomposition of the acetate (decomposition temperature 355 °C [56]) assumedly can be ascribed to effect of acoustic cavitation, the IL might act as a template for a nanorod morphology. This reaction yielding relatively uniform particles with an enlarged band gap compared to the bulk material, might be promising for potential applications [29].

#### 2.1.4. Iron Oxides

For the iron oxides Fe_2_O_3_ and Fe_3_O_4_, different IL-assisted synthesis methods were found. Similar to ZnO and CuO, γ-Fe_2_O_3_ as well as Fe_3_O_4_ were synthesised in the highly hydrated IL [N_4444_]OH from Fe(CH_3_COO)_2_ (100 °C, 10 h). The products were mostly obtained in cubic morphology, although spherical particles were present. According to the authors, this method is quite simple compared to other approaches yielding iron oxide particles of a similar size (8 nm in diameter) [61]. However, as both iron oxides are obtained simultaneously, synthesis optimisation is necessary for practically relevant phase-pure products.

A phase-pure synthesis of α-Fe_2_O_3_ in an ionothermal reaction (180 °C, 12 h) from FeCl_3_·12H_2_O in [pmim]I was reported by Zheng et al. [62]. This reaction also readily takes place in water [15], however, the product was obtained as nanoplatelets assembled to nanoplates. Presumably, the IL neutralises ionic charges at the surface of the nucleus in *c*-direction, thus preventing crystal growth along this plane. The aggregation of the resulting nanoplatelets to regular nanoplates is ascribed to the ability of the IL to form two-dimensional polymeric assemblies. Studies in a mixed solvent of [pmim]I and water showed that the latter disturbs this mechanism, yielding randomly assembled nanoplatelets [62]. 

Furthermore, Yang et al. reported the ionothermal synthesis of γ-Fe_2_O_3_ from Fe(CO)_5_ in the IL [bmim][NTf_2_] (280 °C, 1 h). The reaction, apparently, takes place by the decomposition of Fe(CO)_5_ and the subsequent oxidation to iron(III) by oxygen in the air or its traces in argon atmosphere. Oleic acid added besides acts as a capping agent supporting the formation of nanoparticles with a quite narrow particle size distribution. Nanoparticles of the same quality can also be received by reusing the IL, thus, presenting an applicable way of recycling [63]. 

A microwave-assisted method (2.45 GHz, 900 W) for the synthesis of Fe_2_O_3_ was reported by Gedanken et al. who showed that Fe(NO_3_)_3_ reacts to Fe_2_O_3_ nanoparticles with air oxygen in the IL [bmim][BF_4_]. This is only observed for a short heating period of 2 minutes. After longer irradiation time, the decomposition of the IL takes place involving the reduction of iron(III) to iron(II) by carbon and the formation of FeF_2_ using fluoride from the IL’s hydrolysed [BF_4_]^−^ anion [64].

#### 2.1.5. Cerium Oxide

Similar to the systematic study on TiO_2_ synthesis in ILs, Mudring et al. investigated the formation of CeO_2_, particularly the influence of the cerium precursor, the cation of the IL, the heating method and the precipitator (NaOH, NH_4_OH). Like in the TiO_2_ synthesis, the ILs act as a template while the hydrolysis of the cerium precursors can be ascribed to NaOH or NH_4_OH, respectively [36]. Only ultrasound irradiation (45 kHz, 60 W, RT, 12 h) yielded phase-pure CeO_2_, whereas microwave (2.45 GHz, 80 °C, 10 min) and ionothermal (170 °C, 20 h) heating also produced Ce_2_O_3_ and Ce(OH)_3_ by-products, which can be converted into CeO_2_ via calcination (425 °C, 4 h) [30]. This might be ascribed to the higher temperatures already present in the cavitation bubbles during sonochemical heating. For the sonochemical reaction pathway, an increase in crystallinity of the aggregated nanospheres with increased irradiation time is observed. Using different cerium precursors (Ce(CH_3_COO)_3_, Ce(NO_3_)_3_, CeCl_3_) and ILs has no influence on the phase purity of the CeO_2_ obtained, but, as well as the precipitant, influences the morphology of the nano-sized products. This is ascribed to varying reaction kinetics. Hence, different precursors possess varying hydrolysis rates and, furthermore, the counter ion can influence the morphology by favoured coordination to certain crystal facets. Different IL cations were chosen because of their different potential interactions including a π-system ([bpy][NTf_2_], [edimim][NTf_2_]), an additional acidic proton ([bmim][NTf_2_]), none of both ([bmpyrr][NTf_2_]) or a functional group ([N_1112_OH][NTf_2_]). These different interactions result in different product morphologies with the mechanisms not being clear, yet [30]. This study again shows the huge influence of a wide variety of reaction parameters on the obtained product. 

Using a similar ionothermal approach (150 °C, 2 h), Yu et al. synthesised CeO_2_ nanoparticles in [emim][NTf_2_] with NaOH as precipitator. However, apparently the interactions between the IL and the ceria nanoparticles are relatively high, making calcination (600 °C, 2 h) necessary for the removal of the IL. According to the authors, the resulting CeO_2_ is phase-pure with no evidence for impurities [65]. 

Furthermore, Yan et al. synthesised CeO_2_ from Ce(NO_3_)_3_·6H_2_O in the IL [C_16_mim]Br (100 °C, 2d). Despite ethanol being used for a better dissolution of the starting material, apparently, no additional precipitator is necessary to obtain the oxide. The IL played the role of a solvent as well as a template for nearly monodisperse spherical ceria nanoparticles. The template effect is assumed to be mediated via hydrogen bonding between the IL and cerium hydroxide and π-π-stacking of the imidazolium rings [66].

Mixed oxides of cerium, namely the CO oxidation catalysts Ce_0.5_*M*_0.5_O_2_ (*M* = Ti, Zr, Hf), were synthesised by Mudring et al. in the IL [bmim][NTf_2_] by ionothermal (170 °C, 20 h), microwave (2.45 GHz, 80 °C, 10 min) and sonochemical (45 kHz, 60 W, RT, 9 h) methods. The best catalytic activity of the directly obtained, highly crystalline materials was found for the microwave-heated products. An exchange of the IL’s cation by [P_66614_]^+^ yielded similar spherical nanoparticles. However, using [pmimOH]^+^, which offers significantly stronger interactions via hydrogen bonding, resulted in sheet-like nanoparticle assemblies for Ce_0.5_Zr_0.5_O_2_ [67].

#### 2.1.6. Miscellaneous

For several metal oxides, relatively isolated reports concerning their synthesis in ILs are found. A promising ionothermal method to synthesise γ-Al_2_O_3_ from an IL without the usually necessary annealing step was reported by Zheng et al. Through heating (150 °C, 10 h) of AlCl_3_ in [bdmim]Cl in the presence of sodium amide (NaNH_2_), crystalline nanoparticles of γ-Al_2_O_3_ aggregating to mesoporous nanoflakes were obtained. The IL, in this case, acts as a solvent as well as a template for the worm-like pores. A mechanism is proposed with AlCl_3_ initially reacting to Al(OH)_3_ with NaNH_2_ and traces of water in the IL. Subsequently, Al_2_O_3_ forms under ionothermal conditions. Apparently, in this reaction, Al_2_O_3_ is synthesised at the lowest temperature reported so far [68]. Despite a few research groups describing synthesis routes of Al_2_O_3_ in ILs [69,70,71,72], the one reported by Zheng et al. appears to be the only one yielding alumina without calcination. Therefore, it would be especially interesting to figure out the processes taking place in the IL in future research.

PbO was obtained in a PbS-type crystal structure by Li Juan et al. starting from Pb(OH)_2_ by an ionothermal reaction (180 °C, 3 h) in the IL [beim][BF_4_]. The reaction taking place, merely is the thermal decomposition of Pb(OH)_2_ (decomposition temperature 145 °C [36]), however, the IL acts as template for the crystal structure. So far, no mechanism is suggested for the role of the IL [73].

Tin doped In_2_O_3_ was synthesised in a microwave-assisted approach from SnCl_4_ and InCl_3_·4H_2_O in the IL [N_1444_][NTf_2_] in the presence of DMF and [N_1111_]OH by Feldmann et al. (300 °C, 15 s). The nanocrystalline product was obtained in thin, transparent layers allowing the utilisation as electrode material. Thereby, the IL appears to be crucial for charge stabilisation, thus, avoiding particle agglomeration [74].

The same IL was used for the synthesis of the manganese oxides hydrohausmannite and Mn_3_O_4_ in the shape of nanodisks by Taubert et al. (100 °C, 10 h). However, this mixture of manganese oxides had to be calcined in order to yield phase-pure Mn_3_O_4_. Despite a small amount of water was added in order to improve the solubility of Mn(CH_3_COO)_2_, the authors claim that this can be avoided by the use of other manganese salts than Mn(CH_3_COO)_2_ [61].

[N_4444_]OH was also applied to the ionothermal synthesis of the phase-pure mixed metal oxide SrTiO_3_ by Ruck et al. (180 °C, 20 h). Apparently, at the beginning of the reaction, Ti(O*^i^*Pr)_4_ is hydrolysed to form an amorphous intermediate, which subsequently reacts with strontium(II) cations. Thereby, the morphology of the intermediate is preserved. A specific amount of IL is necessary to achieve the completeness of the reaction, however, in its hydroxide ion providing function, it can be replaced by other bases, e.g., NaOH. Altogether, in this synthesis, particle sizes in the range of 50–150 nm were obtained, whereby the desert-rose-like morphology depends on the presence of ethylene glycol [75].

#### 2.1.7. Conclusion on IL-Based Metal Oxide Synthesis

In so far investigated systems, ILs most commonly act as solvent and template in ionothermal, microwave-assisted and sonochemical reactions that take place in IL-free solvents as well. However, a better control of phase and morphology of the often nanostructured metal oxides is possible. This is due to the interactions between the IL and the metal oxidic nuclei. In most cases, the role of the cation is considered more important as hydrogen bonds as well as π-π-interactions can be formed. However, the effect of the anions, despite just weakly coordinating, should not be neglected.

Understanding some effects of ILs should not hide the fact, that their role and reaction mechanisms, in many cases, are not clarified at all. Previous investigations have been of rather empirical nature, whereas further studies could support models for the prediction of reactions. It should also be taken into account, that the reaction outcome not only depends on the IL, but various factors, such as heating method, reaction temperature and additives, have a significant effect on the product, which is not completely understood, yet. Also, effects of the concentration of the IL, respectively the amount of additives, and the composition of IL mixtures should be investigated in more detail.

The previously discussed synthetic approaches, in many cases, stand out because of their relative experimental straightforwardness. Therefore, the application of ILs could mean a significant simplification for the synthesis of defined metal oxide phases compared to conventional methods. However, it has to be taken into account, that for most applications a separation of the metal oxide particles from the IL is necessary. This can be performed by relatively uncomplicated washing with suitable solvents or in some cases, calcination might be needed. As for nanoparticles, already small traces of adherent IL might change their properties, their purity should always be questioned critically and examined by suitable methods, such as IR, Raman or energy-dispersive X-ray spectroscopy.

Thus, ILs are a promising reaction medium for the synthesis of metal oxide particles in well-defined shapes, but a comprehensive understanding still lacks systematic data.

Furthermore, a promising approach for a completely new reaction pathway for metal oxide synthesis is reported by Zheng et al. who synthesised γ-Al_2_O_3_ at only 150 °C, a significant decrease compared to conventional calcination. Systematic studies could give valuable information about a more efficient synthesis of Al_2_O_3_ and other metal oxides. Especially the mechanism of this ionothermal synthesis is of great interest.

### 2.2. Reactions in DESs

A reaction medium with very similar characteristics and properties to ILs are DESs. Most common DESs are mixtures formed by a quaternary ammonium salt and a hydrogen bond donor [20,76,77]. Characteristic for DESs is the low melting point, which is significantly below the melting points of the individual components. This is illustrated by the example of the very popular nontoxic and cheap choline chloride-urea DES (molar ratio 1:2). While choline chloride and urea melt at 302 °C and 133 °C, respectively, the DES has a melting point of only 12 °C, which is ascribed to the strong hydrogen bonds in the solution [20]. Most approaches for metal oxide synthesis described below were performed in this DES, choline chloride-urea (molar ratio 1:2), taking advantage of the decomposition of urea at temperatures above 100 °C, which produces NH_3_. Apparently, these NH_3_ molecules form intermediate ammine complexes and, thus, play an important role as a template for nanostructures or as a reactant [78,79,80,81,82].

#### 2.2.1. Zinc Oxide

In a DES formed from oleic acid and different alkyl amines, Xie et al. synthesised ZnO micro-pyramids from zinc acetate (286 °C, 1 h). The thermal decomposition of the reagent takes place in non-DES solvents as well forming wurtzite ZnO of irregular or nanorod morphology. However, the DES, apparently, lowers the surface energy of the polar surfaces, hence, supporting the growth of hexagonal micro-pyramids [83].

#### 2.2.2. Iron Oxides

Tu et al. synthesised α-Fe_2_O_3_ from FeCl_3_ in a reaction at 200 °C in choline chloride-urea (molar ratio 1:2). NH_3_ arising from decomposed urea plays an important role in the proposed mechanism to form the complex intermediate Fe(NH_3_)_2_Cl_3_ that reacts with Fe_2_O_3_ after the addition of water. Thereby, Fe_2_O_3_ in the form of nanospindles is obtained [84]. 

A microwave-assisted reaction to Fe_2_O_3_ was applied by Edler et al. heating Fe(NO_3_)_3_·9H_2_O in choline chloride-urea (molar ratio 1:2, 300 W, 100–200 °C, 10 min). Phase, size and morphology of the obtained particles depend on the reaction temperature and the water content. This is attributed to the decomposition of urea depending on both factors. Hence, γ-Fe_2_O_3_ was obtained at 150 °C, but α-Fe_2_O_3_ at 200 °C. Altogether, the addition of small amounts of water was found to have a positive effect, due to a lower viscosity and the supported hydrolysis of urea. [85].

Another iron oxide, Fe_3_O_4_, was synthesised by Chen et al. via co-precipitation in choline chloride-urea (molar ratio 1:2). By the heating of FeCl_3_ and FeCl_2_ (80 °C, 1.5 h), spherical, magnetic Fe_3_O_4_ nanoparticles were obtained. As the product particles are more regular and smaller than the products obtained when the same reaction is carried out in water, the DES apparently acts as a template during the nanoparticle formation. Despite no hydrolysis of urea taking place at the reaction temperature, mostly the choline cations are assumed to be absorbed at the Fe_3_O_4_ particle surface, preventing uncontrolled growth [78]. A method without the decomposition of urea has the advantage of being potentially recyclable, hence, more efficient.

#### 2.2.3. Tin Oxides

Gu et al. synthesised SnO from SnCl_2_ in choline chloride-urea (molar ratio 1:2, 100 °C, 1–60 min). A reaction mechanism is proposed with SnCl_2_, at first, reacting to Sn(OH)Cl with traces of water in the DES. Subsequently, the reaction with ammonia released from urea yields SnO and NH_4_Cl. The SnO particles obtained are nano- and microsized because of the fine dispersion in the solution. Apparently, oxygen in the air causes the oxidation to SnO_2_ [86]. 

Furthermore, they found a method to synthesise SnO_2_/graphene nanocomposites from SnCl_2_ and graphene oxide sheets in an ultrasound-assisted reaction in choline chloride-urea (molar ratio 1:2, RT, 4 h). A mechanism is suggested with graphene oxide being reduced by SnCl_2_ and subsequent SnO_2_ deposition on the graphene sheets. The advantage of this DES reaction medium, according to the authors, is the uncomplicated experimental setup [80]. However, such SnO_2_/graphene composites can also be synthesised in other reaction mediums, like a water-HCl-urea mixture [87].

The group also used N_2_H_4_·H_2_O as additive for the synthesis of nanograined SnO_2_ from SnCl_2_·2H_2_O in the DES choline chloride-urea (molar ratio 1:2, 1 h). A mechanism is proposed with N_2_H_4_·H_2_O reducing tin(II) to tin(0) nanoparticles. The DES, subsequently, prevents the agglomeration of the particles, which allows the reaction with oxygen because of their high reactivity [88].

#### 2.2.4. Miscellaneous

Another DES applied to metal oxide synthesis is choline chloride-ethylene glycol (molar ratio 1:2). With the additional help of acetic acid, Fu et al. synthesised nanosized cuboctahedral particles of tungsten molybdenum oxide composites from (NH_4_)_2_WO_4_ and (NH_4_)_6_Mo_7_O_24_ (40 °C, 6 h). Although no detailed reaction mechanism was proposed, the DES could compensate high surface energies [89]. 

#### 2.2.5. Conclusion on DES-Based Metal Oxide Synthesis

Only a few experiments regarding the synthesis of metal oxides in DESs have been performed, yet. This research focused on the utilisation of DESs as template for more homogeneous nanostructures of oxide materials that could be obtained in DES-free aqueous solutions as well. From the small amount of data on the synthesis of metal oxides in DESs no predictions of reaction results and promising approaches are possible.

Often the formation of NH_3_ from decomposing urea is made use of, which subsequently can act as a reactant. Naturally, the consumption of the DES for a reaction restricts its recyclability. However, because of the lower cost and toxicity of DESs compared with ILs, they might still be valuable reactions systems. Furthermore, a reaction at just 80 °C without the decomposition of urea showed, that the intact components of DESs can be beneficial for reactions, as well. Therefore, this might be a promising approach for future research.

Despite the small amount of previous research, DESs exhibit favourable dissolution properties, which widely differ from molecular solvents [77]. They could more systematically be applied to different kinds of starting materials in order to find new reaction pathways for the synthesis of metal oxides. Especially their lower cost and higher environmental compatibility compared to many ILs could make them an interesting alternative.

### 2.3. Hydroxide Synthesis and Calcination

Another approach for the synthesis of metal oxides in ILs or DES is their utilisation for an efficient synthesis of the respective metal hydroxide in a desired morphology followed by an annealing step. This synthesis principle is not uncommon [69,70,71,72,90], but as the IL, respectively the DES, only takes part in the formation of a precursor and the energy-consuming calcination step still has to be performed, only a few examples are mentioned here. 

Mudring et al. used a microwave-assisted reaction method (2.45 GHz, 80 °C, 10 min) to synthesise SrSnO_3_ from Sr(CH_3_COO)_2_ and SnCl_4_ with different ILs ([bmim][NTf_2_], [C_6_(mim)_2_][NTf_2_]_2_, [bpy][NTf_2_], [P_66614_][NTf_2_]) in the presence of NaOH acting as solvent and template. The obtained SrSn(OH)_6_, subsequently, was calcined (700 °C, 3 h) to yield SrSnO_3_ [91]. The reaction was also successfully performed in an ionothermal synthesis (170 °C, 20 h) before calcination [27]. Despite the reaction takes place in aqueous solution, as well, the IL prevents agglomeration and different cations influence the morphology of the nano-SrSnO_3_. Thereby, different possible interactions ways, such as hydrogen bonding and π-π-interactions, have significant effects, however, the mechanisms are not understood, yet [91]. 

The same reaction method has been applied to Sr_1-x_Ba_x_SnO_3_ (x = 0; 0.2; 0.4; 0.6; 0.8; 1) using Ba(CH_3_COO)_2_ as an additional reactant in the IL [bmim][NTf_2_]. Solid solutions of SrSnO_3_ and BaSnO_3_ without any detectable impurities were obtained [27].

Using a DES, Gu et al. synthesised NiO from NiCl_2_ in choline chloride-urea (molar ration 1:2) at 150 °C (40 min). After the addition of water and workup, the Ni(OH)_2_ precipitation was annealed (300 °C, 4 h). Nano-sized, flower-shaped NiO built from smaller grains with a mesoporous structure was obtained. The effect, which is used in the DES-reaction, is the decomposition of urea, producing NH_3_ that reacts with the well-dispersed water and thereby provides OH^−^ as reaction sites. The experiment was also performed in an aqueous NaOH solution with both reactions yielding phase-pure NiO. As NaOH in the aqueous solution is not as well dispersed, nucleation is far more inhomogeneous [79,81]. Using the same reaction method with slight changes in reaction temperature and time, the research group also obtained Co_3_O_4_ as mesoporous nanosheets [82]. 

Furthermore, Su, Wen et al. produced sheets of Na_2_Ti_3_O_7_, interesting as an anode material for sodium-ion batteries in the DES choline chloride-ethylene glycol (molar ratio 1:2, 80 °C). Despite calcination of the precursor (500 °C, 7 h) being necessary, the reaction temperature could significantly be reduced compared to conventional synthesis [92]. 

Similarly, a γ-CoV_2_O_6_ precursor was synthesised by Seo et al. in the DES choline chloride-malonic acid (molar ratio 1:1, 100 °C, 2 h). The cobalt vanadate was obtained after additional 2 h calcination at 500 °C, which is significantly lower than the conventional solid-state synthesis at 900 °C in 6 h. The authors suggest that the decomposition of the DES generates in situ high thermal conditions at relatively low temperatures leading to the formation of γ-CoV_2_O_6_. Furthermore, the DES, apparently, acts as a template for octahedral nanoparticles [93]. 

The same IL was used by Boston et al. for the synthesis of a barium titanate precursor from barium acetate and titanium isopropoxide. However, the final oxide was obtained after 6-h calcination at 500 °C and 1 h at 950 °C [94]. 

In a similar approach, Zhang et al. used the DES glucose-urea (molar ratio 1:3) as a reaction medium and template for the catalytically active binary oxide Ir_2_SnO_x_ from the chloride salts (180 °C, 12 h). The elevated temperature causes the polymerisation of glucose, hence, the formation of a foam in which the metal ions are embedded. Carbon is finally removed by calcination at 500 °C yielding porous Ir_2_SnO_x_ in nanorod morphology. According to the authors, urea also plays a crucial role by providing coordinating NH_2_ groups which support a uniform composition of the product [95]. At the temperatures used this could rather be NH_3_ ligands.

König et al. synthesised spinel-type ferrite nanoparticles *M*Fe_2_O_4_ (*M* = Mg, Zn, Co, Ni) by dissolving equimolar amounts of Fe_2_O_3_ (haematite) and *M*O (*M* = Mg, Co, Ni, Zn) in ten different DESs. They consisted of choline chloride, urea or *N*,*N*′-dimethylurea as hydrogen bond acceptors and organic acids (maleic, malic, citric acid), sugars (d-mannose, d-fructose, d-glucose), *N*,*N*′-dimethylurea or vanillin as hydrogen bond donors. In the first step, the dissolution was performed in two hours at 80 °C, subsequently, the ferrite nanoparticles were obtained via calcination at 400–600 °C. Despite calcination being necessary to obtain the product, the reaction temperature was lowered compared to conventional solid state reactions at 900 °C [96]. No reaction mechanism was suggested, however, all DES components contain O and N atoms suitable to coordinate to metal ions, thus supporting the dissolution of the starting materials.

Even if ILs and DES, in several systems, do not give metal oxides as a product, but only the hydroxide precursor, such methods can be beneficial compared to IL- or DES-free approaches. Thus, in many cases the calcination temperature can be lowered, which at least is an auspicious beginning for reducing the amount of energy needed. Furthermore, the interactions of the solvent influence the morphology of the metal hydroxide, which, usually, is preserved in the calcined metal oxide, as well. Thus, ILs and DESs, in these systems, also exhibit a template effect. As many pure metal oxides could be obtained in different phases and morphologies directly in ILs and DESs, the hydroxide calcination approach seems to be rather promising for mixed metal oxides, where less research has been performed, yet.

## 3. Dissolution of Metal Oxides

### 3.1. Dissolution in ILs

The processing of metal oxides, present in naturally occurring ores, earths and minerals as well as in (nuclear) waste, with conventional energy- and resource-demanding methods is considered increasingly problematic. In order to find solutions for the disadvantages in these processes, such as high temperatures, large amounts of waste and toxic, volatile solvents, ILs appear to be a promising reaction medium. 

**Figure 2:**
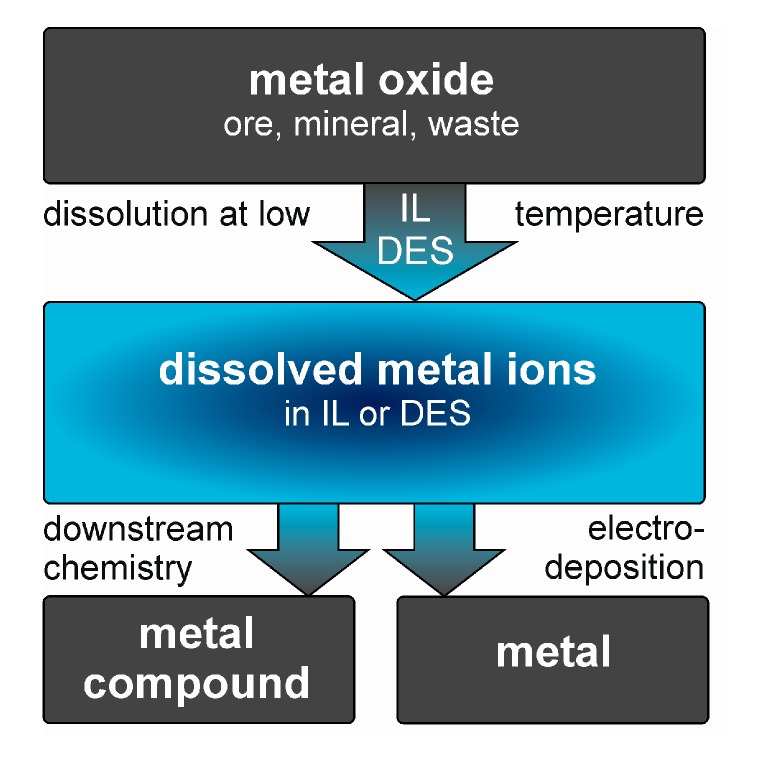
Scheme of the general idea of metal oxide processing in ILs and DESs.

The underlying idea of the application of ILs and DESs for metal oxide processing is a two-step process, as shown in Figure 2. First, metal oxides are dissolved in the IL or DES at relatively low temperatures. Second, proceeding from the obtained solution, either metal compounds are synthesised via downstream chemistry or metals are electrochemically deposited. Regarding the sustainability of such processes, the recyclability of the ILs is highly desirable. Despite a lot of research has been performed on metal extraction using ILs, a large number of these reports assumes metal ions dissolved in and extracted from different solvents [97,98,99,100]. The direct dissolution of metal oxides by ILs, however, appears to be considerably less investigated. Despite the topic was addressed in some review articles [22,23], it was covered rather secondarily. A more comprehensive summary on the dissolution of metal oxides in ILs is given in the following section. Detailed information about the dissolution of specific metal oxides in different ILs is listed in Table 2.

#### 3.1.1. Chloridometalate ILs

First experiments on the dissolution of metal oxides have been performed in chloridoaluminate ILs, consisting of a chloride salt with a large organic cation and AlCl_3_. Depending on the molar ratio of these components, basic, neutral and acidic ILs are distinguished. The IL is considered basic with an excess of the organic chloride salt and acidic with an excess of AlCl_3_, while neutral ILs consist of an equimolar amount of both, i.e., [AlCl_4_]^−^. Because of the hygroscopy of AlCl_3_, all experiments have to be performed under inert gas atmosphere [19].

Dissolution experiments have especially been performed in Lewis-basic ILs, as the presence of free chloride anions appears important for the formation of soluble complex anions. Thus, Dai et al. dissolved UO_3_ in basic ILs of [emim]Cl or [pdmim]Cl and AlCl_3_. Furthermore, a connection between the ability of the IL cation to form hydrogen bonds and the solubility of UO_3_ can be drawn, as higher solubilities are found in the [emim]^+^ IL with a hydrogen atom at C2. The favoured formation of the stable [UO_2_Cl_4_]^2−^ complex anion [118] was shown by molecular dynamics studies by Chaumont and Wipff [123].

A wider range of Lewis acidity was covered by the studies of V_2_O_5_ in acidic, neutral and basic [emim]Cl/AlCl_3_ and [bmim]Cl/AlCl_3_ by Bell and Castleman. They reported the formation of VO_2_Cl_2_^−^ and metavanadates [(VO_3_)*_n_*]*^n^*^−^ (*n* = 3, 4) as main species with their ratio being dependent on the acidity of the IL and the concentration of V_2_O_5_. Furthermore, in acidic melts, the reaction of these species with Al_2_O_7_^−^ is assumed, yielding volatile VOCl_3_. In contrast to that, no dissolution was observed for ILs with weakly-coordinating anions, such as [bmim][BF_4_] and [bmim][OTf]. This highlights the importance of strong interactions between the IL and the metal oxide, necessary to overcome the lattice energy of the solid [121]. 

V_2_O_5_ as well as Bi_2_O_3_ were also dissolved in the neutral IL [bpyr]Cl/AlCl_3_ by Mahjoor and Latturner. Upon dissolution, the chlorination of the metal atoms takes place yielding the complex anions [V_4_O_4_Cl_12_]^4−^ and [Bi_4_Cl_16_]^4−^ [107]. 

Shi et al. dissolved Li_2_O in the Lewis-basic IL ethylene carbonate/AlCl_3_. They suggest the strong coordination power of the hypothetical [AlCl_2_]^+^ cation as driving force for the dissolution of Li_2_O. Differing from the previous ILs, instead of tetrachloridoaluminate, in this IL, [AlCl_2_(ethylene carbonate)_4_]^+^ complexes are formed. Subsequently, the ligand exchange between ethylene carbonate and Li_2_O causes the dissolution [112].

Similar to aluminium, also iron appears to be a suitable addition to an IL for the dissolution of UO_2_, as Yao and Chu showed. They reported the oxidation, hence dissolution, of the metal oxide to UO_2_^2+^ in mixtures of imidazolium-based, iron-containing ILs and their corresponding imidazolium chloride IL. In the course of this, [FeCl_4_]^−^ is reduced to [FeCl_4_]^2−^ [115]. This implies that the same IL cannot be reused for the dissolution of UO_2_ without previous oxidising workup.

Deducing from these reports, chloridometalate ILs can be suitable solvents for the dissolution of metal oxides as they provide strong interactions to the metal atom of the metal oxide. In most cases, chloride ions play a vital role as strong ligands in the resulting coordination sphere. However, a large drawback of this kind of IL is their water and air sensitivity, requiring expensive inert gas atmosphere set-ups and complicating up-scaling.

#### 3.1.2. Air- and Water-Stable ILs

In order to overcome the disadvantages of chloridometalate ILs, further research on the dissolution of metal oxides in ILs focused on the application of different ILs with discrete anions. They allow an experimental set-up under ambient conditions and the application of aqueous additives. As such anions often are merely weakly coordinating, the interactions necessary for the dissolution of metal oxides, are provided by the cations in most cases.

As the oxygen anion of a metal oxide is too unstable to exist in a free form, an obvious approach could be its transfer into stable compounds, thereby dissolving the metal ions. Hence, protons could be provided for the reaction to water. In water-stable ILs, aqueous acids can easily be added for this purpose, as Binnemans et al. showed. They dissolved CaO, CoO, CuO, Fe_2_O_3_, MnO, NiO and ZnO in the acid-saturated IL [P_66614_]Cl (10 wt % 12 M HCl), whereas the respective tetrachloridometalate anions formed, except for calcium, where a partly hydration is assumed. Indeed, by this method metal oxides were directly dissolved into an IL phase, however, the solubility is similar to pure hydrochloric acid [108].

Similarly, Billard et al. dissolved Eu_2_O_3_, Nd_2_O_3_, Pr_6_O_11_, UO_2_ and UO_3_, in a mixture of [bmim][NTf_2_] and small amounts aqueous nitric acid (14 M). For uranium, UO_2_(NO_3_)_3_^−^ was identified as an ionic species present in the solution, revealing the oxidation of UO_2_ by the reduction of NO_3_^−^ to NO_2_^−^. They, furthermore, reported a reaction-promoting effect of water, as the dissociation of nitric acid might be supported [111].

Instead of adding mineral acids, Moyer et al. used the acidic IL [dmah][NTf_2_] for the dissolution of UO_3_. The [dmah]^+^ cation is assumed to dissociate in a proton and the corresponding base which is suitable to solvate the uranyl ion. Apparently, the water evolving during the reaction has a reaction-promoting effect by supporting the dissociation of [dmah]^+^. [emim][NTf_2_] turned out to be a suitable inert additive for dilution in order to lower the viscosity for subsequent electrochemical experiments [119]. Despite the IL cation directly acting as a reactant in the reaction, it should be possible to recover it by addition of an acid, hence protonation. Thus, the metal ions could be transferred to another phase and the IL recycled.

Similarly, Binnemans et al. made use of the acidic proton at the C2 position in imidazolium cations. Thus, they demonstrated the solubility of Ag_2_O, CuO, NiO and ZnO in several imidazolium-based ILs via the coordination of carbene ligands. The deprotonation of C2 and the subsequent carbene formation appear to be the driving force of the metal oxide dissolution [101], as a suitable ligand for the metal cation is provided.

Also using the coordinating abilities of the IL’s cation, Endres et al. applied the IL [mim][OTf] for the dissolution of ZnO. Zinc(II) is assumed to be chelated by 1-methylimidazole via the nitrogen atoms. Furthermore, Zn–O interactions are proposed with the [OTf]^−^ anion as well as with water molecules [122].

Applying a rather uncommon anion, Zarzana et al. dissolved UO_2_ and UO_3_ in the IL [emim][F(HF)*_n_*] (*n* = 2, 3). This IL exhibits a significant fluorinating ability, promoting the dissolution of metal oxides. The dissolved species for UO_2_ is assumed to be UF_6_^−^ at first, which is further oxidised to uranium(VI) by oxygen from air. Upon this process, the diffusion of oxygen into the solution takes place, yielding uranyl fluoride species [116]. This IL should rather be perceived as a fluorinating agent then as a solvent for metal oxides, as the dissolution process involves the consumption of the IL anion. Hence, recyclability is limited.

Using air- and water-stable ILs, naturally simplifies experimental works. However, the strong interactions to the metal oxide’s cation, which tetrachloridometalate ILs offer, have to be provided in another way, in order to dissolve metal oxides. Mostly, this is achieved by cations featuring electron-rich atoms acting as ligand. Furthermore, it appears reasonable to provide protons for the reaction with oxygen. This could be a driving force for the reaction, according to Le Châtelier’s principle.

#### 3.1.3. Task-Specific ILs

**Figure 3:**
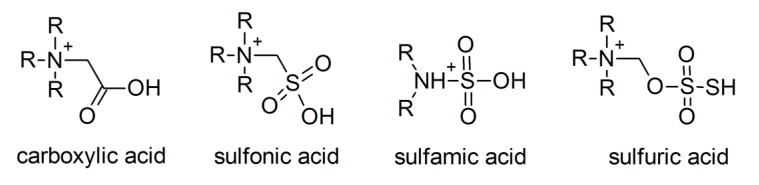
Overview of Brønsted acidic task-specific ILs (TSIL) cations. The picture shows the simplest representative of each kind of IL as variation in the length of the alkyl chain between the ammonium and the acidic functional groups are possible.

Large progress in the dissolution of metal oxides was made by the application of task-specific ILs (TSILs), featuring a functional group, a carboxyl group, at the cation. Brønsted-acidic groups combine both advantages already attempted in common water-stable ILs: On one side, protons are suitable to form water with the oxygen atom of the metal oxide, on the other, the remaining, electron-rich functional group is a suitable ligand for the metal ions. Figure 3 gives an overview about the Brønsted acidic TSIL discussed in this review.

**Figure 4:**
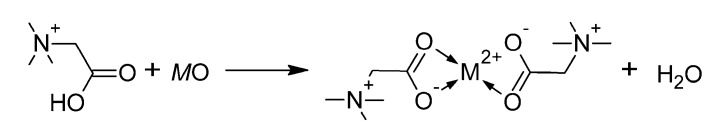
Dissolution mechanism of a metal oxide in [Hbet][NTf_2_].

As an example for carboxyl-functionalised ILs, a lot of research has been performed using the IL [Hbet][NTf_2_], first described by Binnemans et al. [102]. After the water-forming reaction of acidic betainium protons with oxide atoms from the metal oxide, the remaining betaine molecule coordinates to the metal cation to form metal-betaine complexes, as shown in Figure 4. 

As betaine is too sterically demanding to saturate all coordination sites, smaller molecules are necessary in order to promote dissolution. Therefore, all reactions were performed in aqueous solutions, thus providing aqua ligands. Although numerous crystal structures of polymeric metal-betaine-aqua complexes were described [102,103], synchrotron techniques revealed a dissociation to the monomeric complexes in solution [114]. A large number of oxides of main and subgroup as well as lanthanide and actinide metals were dissolved in aqueous [Hbet][NTf_2_] [18,102,103,113,114,117,124,125], however, negligible dissolution was observed for not readily soluble, more covalent metal oxides, such as Al_2_O_3_ [102,126,127], Fe_2_O_3_ (haematite) [126,127], MnO_2_ [102], TiO_2_ (anatase-rutile) [127], cobalt oxides [102] and U_3_O_8_ [117]. 

Furthermore, Nockemann et al. and Binnemans et al. reported several TSILs similar to [Hbet][NTf_2_] with a carboxyl group at the cation and a [NTf_2_]^−^ anion suitable for the dissolution of metal oxides [103,120]. For another aqueous carboxyl functionalised IL, 3-(5-carboxypropyl)-1-methylimidazolium bromide, Li et al. found a good solubility of Eu_2_O_3_ [128].

Gupta, Chandrakumar et al. showed that the addition of water as a solvent is not necessary in order to obtain metal-betaine-water complexes, but the water evolving during the reaction is sufficient when refluxed [18]. 

It has to be taken into account, that some metal oxides undergo redox reactions upon dissolution. This might involve a partly decomposition of the IL. Chen et al. found evidence for the reduction of Pb^4+^ from PbO_2_ to Pb^2+^ in [Hbet][NTf_2_]. The same complex [Pb(bet)_2_(H_2_O)_2_]^2+^ is formed from PbO_2_ and PbO [113]. For the dissolution of UO_2_, Nagarajan et al. identified the oxidation to UO_2_^2+^ as crucial [117]. 

Furthermore, aqueous [Hbet][NTf_2_] was applied to natural mixtures of substances for the leaching of specific metals from recycling waste. Thus, Davris et al. leached rare earth metals from bauxite residues. A good selectivity for rare earth metals, and, therefore, the dissolution of their oxides, was found against iron (haematite), aluminium, titanium (anatase, rutile) and silicon. Also scandium showed a minor solubility [127]. 

An optimisation of the scandium leaching conditions in this IL regarding the reaction temperature and the molar ratio of the reagents was performed by Mawire and van Dyk [129]. Furthermore, Grimes et al. found that by changing the reaction temperature and the IL-water ratio of aqueous [Hbet][NTf_2_], the relative solubility of rare earths can be adjusted, thus enabling the separation of light (Eu_2_O_3_, La_2_O_3_, Nd_2_O_3_) from heavy rare earths (Y_2_O_3_, Yb_2_O_3_) and Gd_2_O_3_[130]. 

[Hbet][NTf_2_] was also employed for the recycling of fluorescent lamp phosphor waste by Dupont and Binnemans. They selectively dissolved europium doped Y_2_O_3_ as the IL, in contrast to mineral acids, is not able to efficiently dissolve non-oxygen anions [131].

The group of Binnemans also used aqueous [Hbet][NTf_2_] to leach neodymium, cobalt and dysprosium from roasted NdFeB magnets at 80 °C, while iron (haematite) remained almost completely undissolved [124]. 

These examples demonstrate that, on one side, the dissolution characteristics of [Hbet][NTf_2_] can be utilised for specific tasks. Thus, the negligible dissolution of non-oxidic salts as well as covalent oxides, such as Al_2_O_3_, Fe_2_O_3_, TiO_2_ and SiO_2_, enables the separation of metal oxides. On the other, it becomes apparent that the ability of [Hbet][NTf_2_] to dissolve metal oxides is tuneable by varying reaction parameters. Further investigations in this field could reveal new, efficient ways for the separation of metal oxides from starting materials of diverse composition. 

The leaching experiments from NdFeB magnets by Binnemans et al. were repeated in water-free [Hbet][NTf_2_] at 175 °C in a system open to the air, in order to allow the evaporation of water [132]. The absence of water could be advantageous for the further processing of the dissolved metal ions: On one side, it might prove difficult to leach the metal cations from their aqua-complexes for downstream chemistry, on the other, the presence of water might complicate a subsequent electrolysis of the dissolved metals. However, in the approach of Binnemans et al. a significant increase in the reaction time as well as a decrease in the coordination number of the metal ions was found. Because of the steric demands of betaine, not all coordination sites can be occupied. However, a first experiment showed, that chloride as additional, smaller ligand promotes the dissolution of CuO in the absence of water [132].

**Figure 5:**
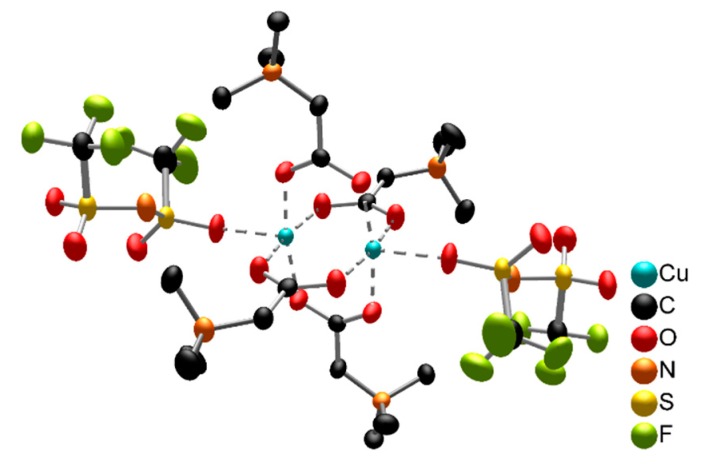
The water-free metal-betaine complex [Cu_2_(bet)_4_(NTf_2_)_2_]^2+^ obtained by the dissolution of CuO in [Hbet][NTf_2_]. Coordinative interactions are marked as dotted lines. The ellipsoids enclose 70% of the probability density of the atoms at 100 K. H atoms are omitted for clarity.

Pursuing this water-free approach, we recently published a comprehensive investigation on the dissolution of 30 metal oxides in water-free [Hbet][NTf_2_]. For the example of CuO, the crystal structure of the water-free copper-betaine complex was solved, as shown in Figure 5. Apparently, in the absence of stronger small ligands, free coordination sites can be occupied by the weakly coordinating [NTf_2_]^−^ anion. Indeed, a positive effect of water on the dissolution was confirmed, but by the addition of [Hbet]Cl, thus providing chloride as additional, nucleophilic ligand in the IL [Hbet]_2_[NTf_2_]Cl, significantly more metal oxides can be dissolved in the IL, including quite unreactive metal oxides, such as Fe_2_O_3_ (haematite), MnO_2_ (pyrolusite) and ThO_2_. However, under the chosen conditions, chloride ions cause a partly decomposition of the IL [106]. Similar to the previously discussed separation experiments, the optimisation of the reaction parameters, such as reaction temperature and duration, as well as chloride concentration could enable the avoidance of this effect. 

While the so far mentioned studies were of rather empiric nature, Qin et al. previously had suggested an *U*/*x* value of a metal oxide *M*_x_O_y_ (*U* = lattice energy) to predict the solubility of metal oxides. This theory was based on dissolution experiments with numerous f-block elements as well as divalent metal oxides. It underlines the correlation between the lattice energy of a metal oxide and its solubility [125]. However, our findings show that this assumption is not valid for all metal oxides, which is in agreement with the report by Gupta, Chandrakumar et al. who dissolved PuO_2_ [18], despite predicted insoluble by Qin et al. [125]. Apparently, the lattice energy is not the only factor influencing the solubility, but also other parameters, such as reaction conditions [18] and crystal phase are important. As the dissolution of metal oxides in [Hbet][NTf_2_] comes down to an acid-base reaction, we consider especially the basicity of the metal oxide to be significant. Hence, for example, a very good solubility is found for alkaline earth metal oxides [106]. However, because of the lack of quantitative data about the basicity of different metal oxides, a validation of this assumption and the exact prediction of dissolution behaviour is not possible, yet.

Making inert metal oxides accessible for dissolution, more acidic IL cations with a sulfonic acid functional group could by applied, as Binnemans et al. reported. Thus in a 1:1 mixture of different sulfonic acid functionalised ILs and water, stoichiometric amounts of cobalt oxides, Fe_2_O_3_ and smaller amounts of Al_2_O_3_, Cr_2_O_3_, TiO_2_ and WO_3_ were dissolved at 80 °C [105]. Other ILs with sulfamic or alkylsulfuric acid functional groups at the cation show a similar or even higher acidity and some dissolution ability for rather unreactive metal oxides [109,110]. Furthermore, the addition of [emim]Cl was found to support the dissolution because of the good solvating ability of the chloride ions [110].

The application of Brønsted-acid-functionalised ILs could make numerous metal oxides accessible for dissolution. Even rather inert metal oxides could be dissolved in increasingly acidic ILs, highlighting the importance of understanding the nature of the acid-base reaction taking place. Several studies of reaction parameters also show, that no absolute statements about an IL dissolving a metal oxide are possible. Instead, always reaction conditions and the composition of the sample have to be taken into account. However, the underlying causes for these effects are not understood, yet. Systematic studies could help to clarify the processes taking place. An advantage of such Brønsted-acidic ILs is that they allow the recycling of the IL. Because of the corresponding base acting as ligand coordinating metal ions, the IL cation is easily retrievable by protonation while the metal ions are leached into another phase.

#### 3.1.4. Conclusion on Metal Oxide Dissolution in ILs

The ability of ILs to dissolve metal oxides results from the coordination abilities of either the cations or anions. Strong nucleophiles, such as chloride, fluoride, carbene or chelating functional groups are suitable ligands. However, task-specific ILs with Brønsted acidic functional groups appear to be especially suitable for the dissolution of metal oxides. The underlying cause is the driving force of the reaction of the protons with the oxygen atoms forming water. The resulting negatively charged functional group, subsequently, is able to act as a chelating ligand, thus, providing relatively strong interactions to the metal cations. Hence, even not readily dissolvable metal oxides exhibit some solubility in these ILs.

Despite one IL fluorinating metal cations via the anion and tetrachloridometalate ILs, dissolution mechanisms appear to sum up to coordinating interactions between the IL cation and metal ions. Anions rather influence these reactions by providing the IL with suitable properties, such as a low melting point, a low viscosity and, possibly, stability towards air and moisture. Especially the very weakly coordinating, bulky anion [NTf_2_]^−^ is used in most ILs for dissolution studies, thus relying on strongly interacting cations. For the example of [Hbet][NTf_2_], other common anions give compounds with melting points well above 100 °C and, therefore, per definition no ILs. However, the complex compound [Cu_2_(bet)_4_(NTf_2_)_2_]^2+^ shows, that this anion can also act as a ligand in the metal ion’s coordination sphere if no stronger ligands are present.

An interesting connection between the acidity of the IL cation and the ability to dissolve not readily soluble metal oxides becomes apparent. The development of new ILs with special regard to the acidity might allow the dissolution of even more metal oxides. On the other side, the dissolution of metal oxides seems to be tuneable by changing the reaction parameters, which is another promising starting point for systematic future research. 

Furthermore, by varying reaction parameters as well as by the application of different ILs, new processes for the segregation of different metal oxides at low temperatures and with smaller amounts of waste might be achievable. In order to design economical as well as sustainable processes, further research should also focus on the recyclability of the IL. Several reports have shown, that the recycling of the IL is possible, next steps should include the design of efficient, maybe even up-scaled processes.

### 3.2. Dissolution in DESs

Some amount of work on the dissolution of metal oxides has also been performed in DESs, although research seems to have had a focus on ILs. Featuring the advantages of lower costs and less toxicity because of various naturally available components, the dissolution process appears to add up to the same principle like in ILs; the presence of coordinating ligands that are able to interact with the metal cations. Nevertheless, there are mechanistic ambiguities that should be resolved by further research. An overview about the metal oxides dissolved in different DESs is given in Table 3.

#### 3.2.1. Choline Chloride-Urea

Early dissolution experiments were performed in the popular DES choline chloride-urea (molar ratio 1:2) by suspending the reagents at temperatures below 100 °C in order to avoid the decomposition of urea. The driving force of dissolution is the coordination of urea to the metal atom [135,140]. This is supported by DFT calculations performed by Rimsza and Corrales. They showed that the favoured complex for CuO is [Cu(urea)]^2+^ with urea in the neutral molecular state [151].

For several easily soluble metal oxides, such as copper oxides [136,137], PbO [142,143,144] or ZnO [137,146,147,148,149,150], various DESs appear to be excellent solvents. Hence, their dissolution, to date, is kind of a chemical routine during the preparation of samples for electrochemical experiments.

Furthermore, Davies et al. found a medium solubility for, MnO_2_ and NiO, but negligible solubility for Al_2_O_3_, CaO and iron oxides. Despite, interestingly, some dissolution was found for the rather inert MnO_2_, an authentic sample of steel furnace waste showed that because of the significantly better solubility of zinc and lead oxides, their selective dissolution is possible [135].

Furthermore, Huang and Zhang showed the dissolution of Ni_2_O_3_ [141], Al-Hajri et al. dissolved MoO_3_ [140] and Antal et al. reported a high solubility of V_2_O_5_, yielding the high-valent, stable [H_2_V_10_O_28_]^4−^ complex anion. Because of a distinct hydrogen bond network, the slow crystallisation of the compound [(CH_3_)_3_N(CH_2_)_2_OH]_4_[H_2_V_10_O_28_]·2(NH_2_)_2_CO occurs within several months [145].

Indeed, urea is able to coordinate to metal atoms, however, usually it acts as a monodentate ligand via the oxygen atom, thus, not offering very strong interactions. However, the few experiments performed with this DES do not allow any general conclusions.

#### 3.2.2. Acidic DESs

Further studies with regard to the importance of the acidity of DESs were performed in mixtures of choline chloride and organic acids below 100 °C. Abbott et al. dissolved CuO, Fe_3_O_4_ and ZnO, in DESs of choline chloride and malonic, oxalic or phenylpropionic acid. All oxides were dissolved to a significant extent, however, the solubility varies in the different DESs without any cause being identifiable [138]. 

In another study, the group showed that malonic acid-based choline chloride DESs (molar ratio 1:1) have a higher solubility of metal oxides than the ones based on urea and ethylene glycol (molar ratio 1:2). This is attributed to the presence of protons acting as oxygen acceptor and, thus, promoting dissolution. Again, the correlation showed that more ionic oxides show a higher solubility in the examined DESs, while the dissolution of more covalent oxides is negligible. However, for almost all investigated metal oxides, the dissolution in aqueous HCl is higher [133]. Comparing the quantitative solubilities of metal oxides in different reports, e.g., [133] with [135] and [138], a significant deviation becomes apparent. Certainly, a variation of the reaction temperature by 10 K will influence the solubility. However, quantitative solubility data should always carefully be examined regarding its accurateness, as the experimental procedure, sample preparation as well as measuring method are sensitive to errors.

In order to determine the effect of the acidity of DESs on the dissolution of metal oxides, Mu et al. investigated the various DESs consisting of different alcohols (ethylene glycol, 1,2-propanediol, glycerol and1,4-butanediol) and various organic acids (malonic, succinic, citric, maleic acid, molar ratio 4:1) at 60 °C. The best performance was found for the most acidic DES ethylene glycol-maleic acid. Furthermore, the deprotonation of the acid upon dissolution of a metal oxide was shown. This DES is suitable to separate light rare earths (≤gadolinium, except cerium) from heavy ones. However, for two examples tested, Eu_2_O_3_ and La_2_O_3_, the solubility was slightly lower than in aqueous hydrochloric acid (3.27 M) [139].

A more acidic DES consisting of *p*-toluenesulfonic acid and choline chloride in varying molar ratios was found by Binnemans et al. They reported a higher solubility of all metal oxides than in previously investigated DESs except for PbO. MnO_2_, Fe_2_O_3_, Fe_3_O_4_ and CuO dissolved better in the DES than in aqueous HCl of a similar pH value. Furthermore, different molar ratios of the DES components appear to influence the dissolution of the metal oxides in different ways, opening possibilities for the development of highly selective leaching processes. Interestingly, usually the most acidic IL does not show the best solubility for metal oxides [134].

Furthermore, DESs were applied to the recycling of authentic mixed metal oxide samples. Thus, Binnemans et al. performed studies on the leaching of neodymium, dysprosium, praseodymium and gadolinium from roasted NdFeB magnets by three different DESs. Choline chloride in mixtures with twice the amount of either urea or ethylene glycol only showed a poor dissolution of iron and neodymium oxide. A significantly better performance was found for choline chloride-lactic acid (molar ratio 1:2), attributed to the presence of acidic protons. Furthermore, the coordinating abilities of lactate or choline are assumed to have a positive effect [152]. 

In order to recycle the cathode of lithium ion batteries, Ajayan et al. investigated the solubility of LiCoO_2_ by using the DES choline chloride-ethylene glycol (molar ratio 1:2). A beginning extraction of cobalt was found at 80 °C, whereby the extraction efficiency significantly increased with higher reaction temperatures (investigated up to 220 °C) and longer reaction times, hence, being comparable to phosphoric and hydrochloric acid. Apparently, during dissolution, cobalt(III) is reduced to cobalt(II) which might involve the oxidation of ethylene glycol. However, an electrodeposition of cobalt allows the reuse of the DES in additional cycles. The leaching efficiency of lithium for LiCoO_2_ exceeds the one of cobalt [153].

Using Brønsted-acidic DESs, improves the solubility of metal oxides. This is attributed to the same effects like in ILs; the water-forming reaction of the acidic protons with the oxygen atoms of the metal oxides has a dissolution-supporting effect. Subsequently, the carboxylate group can build up adequate interactions to the metal ion via coordination. However, only in a few cases, the solubility in a DES exceeds the one in mineral acids. In this regard, the investigation of DESs with a sulphuric acid functionalised component might be promising. But even the so far investigated DESs could be interesting as a replacement of mineral acids in order to avoid aqueous waste in conventional metal recovery processes.

#### 3.2.3. Conclusion on Dissolution of Metal Oxides in DESs

In recent years, less research has been performed concerning the dissolution of metal oxides in DESs than in ILs. Similar to ILs, the availability of coordinating ligands appears to be important for the dissolution of metal oxides. Furthermore, the presence of acidic protons as oxygen acceptors supports dissolution. However, the dissolution ability does not always seem to correlate with the acidity of the DES. Hence, other, yet not identified factors are assumed to have an effect. Further work is necessary to reveal the dissolution mechanisms and dissolved species present in the recently investigated systems. 

The varying dissolution ability of different DESs could be used for selectively dissolving metals from oxide mixtures. The lower synthetic effort and costs compared to ILs might prove advantageous for future applications. Even if the dissolution ability might not reach the same degree as ILs, DESs could be a valuable part of dissolution and extraction processes. By the replacement of aqueous solvents, large amounts of waste might potentially be avoidable, if DESs can efficiently be recycled.

## 4. Conclusions

In the past two decades, research on the utilisation of ILs and DESs in the synthesis of metal oxides clearly focused on the production of nanoparticles. Typically, these reactions can be performed in other media as well, however, certain morphologies and phases could be made accessible and improvements towards the conventional methods were found. Therefore, ionothermal, microwave- as well as ultrasound-assisted methods seem to be suitable. However, in many cases, the underlying mechanisms and the structure-directing role of the IL are not clear, yet. Thus, further investigations in new systems still rather rely on educated guesses instead of allowing predictions and synthesis planning underpinned by assured knowledge. Therefore, future research should focus on getting insight into the reactions taking place on molecular level in order to understand and use the full potential of ILs and DESs. Besides, the synthesis of Al_2_O_3_ at a temperature as low as 150 °C reported by Zheng et al. highlights ILs as a promising solvent for new low-temperature reaction pathways for metal oxides. Again, further research should identify the reaction mechanism in order to apply this approach on larger systems as well as other metal oxides.

The research on the dissolution of metal oxides identified several ILs and DESs suitable to dissolve different metal oxides to a variable extent. Ongoing research on new systems produced ILs that were increasingly able to dissolve not readily soluble metal oxides, whereas a strong correlation with their acidity became apparent. Therefore, it seems possible that in the future, suitable ILs for the dissolution of every metal oxide will be found. The different dissolution ability appears to be a promising property for the selective leaching of metals from ores as well as waste. Despite DESs often showing no better solubility for metal oxides than conventional aqueous solvents, they should be considered as low-cost alternative to ILs, where possible. Especially for the selective leaching of metal oxides from waste, ILs as well as DESs might be used together in optimised processes. In future research, also downstream chemistry proceeding from such metal oxide solutions should gain centre stage. Thus, suitable methods for the synthesis of chemicals in demand as well as the electrodeposition of metals should be developed. 

New syntheses of metal oxides as well as new metal oxide processing paths without calcination steps and other high-temperature reactions could mean great energy savings and would be a step towards a greener future. Completely new reaction pathways could have impact on industrial processes. However, because of the high costs of some ILs, it will be important to recycle and reuse them. 

Altogether, the research field of metal oxide chemistry in ILs and DES still seems to be at its beginning. A lot of further research will be necessary in order to understand the processes taking place, apply them to desired systems and maybe even bring them to industrial relevance.

## Figures and Tables

**Table 1:** Synopsis of used abbreviations of cations and anions of Ionic liquids (ILs) as well as other reagents in the review.

Abbreviation	Full Name
[amim]^+^	1-allyl-3-methylimidazolium cation
[bdmim]^+^	1-butyl-2,3-dimethylimidazolium cation
[beim]^+^	1-butyl-3-ethylimidazolium cation
bet	betaine
[bmim]^+^	1-butyl-3-methylimidazolium cation
[bmpyrr]^+^	1-butyl-1-methylpyrrolidinium cation
[bpy]^+^	1-butylpyridinium cation
[C_6_(mim)_2_]^2+^	6-*bis*(3-methylimidazolium-1-yl)hexane cation
[C_16_mim]^+^	1-hexadecyl-3-methylimidazolium cation
[dca]^−^	dicyanamide anion
[dmah]^+^	N,N-dimethylacetamidium cation
[edmim]^+^	1-ethyl-2,3-dimethylimidazolium cation
[emim]^+^	1-ethyl-3-methylimidazolium cation
[F(HF)_n_]^−^	fluorohydrogenate anion
[Hbet]^+^	betainium cation
[mim]^+^	1-methylimidazolium cation
[N_1112_OH]^+^	2-hydroxyethyltrimethylammonium cation
[N_111_C_2_OSO_3_H]^+^	trimethylammoniumethane hydrogen sulfate cation
[N_4444_]^+^	tetrabutylammonium cation
[N_1888_]^+^	methyltrioctylammonium cation
[N_RRR_C_3_SO_3_H]^+^	trialkylammoniumpropanesulfonic acid cation
[N_RR_HSO_3_H]^+^	dialkylsulfamic acid cation
[NTf_2_]^−^	bis(trifluoromethylsulfonyl)imide anion
[OAc]^−^	acetate anion
OBu^−^	butoxide
O^i^Pr^−^	isopropoxide
[OTf]^−^	trifluoromethanesulfonate anion
[P_66614_]^+^	trihexyltetradecylphosphonium cation
[P_RRR_C_3_SO_3_H]^+^	trialkylphosphoniumpropanesulfonic acid cation
[pdmim]^+^	1-propyl-2,3-dimethylimidazolium cation
[pmim]^+^	1-propyl-3-methylimidazolium cation
[pmimOH]^+^	1-(3-hydroxypropyl)-3-methylimdazolium cation

**Table 2:** Overview about metal oxides dissolved in different ILs as well as corresponding references.

Metal Oxide	Solvent	Reference
Ag_2_O	[bmim]Cl	[101]
	[emim][SCN]	[101]
	[emim][dca]	[101]
	[emim][OAc]	[101]
	[Hbet][NTf_2_]/H_2_O and derivates	[102,103,104]
Al_2_O_3_	[NRRRC_3_SO_3_H][NTf_2_]/H_2_O	[105]
	[PRRRC_3_SO_3_H][NTf_2_]/H_2_O	[105]
BaO	[Hbet][NTf_2_]	[106]
	[Hbet]_2_[NTf_2_]Cl	[106]
Bi_2_O_3_	[bpyr]Cl/AlCl_3_	[107]
	[Hbet][NTf_2_]	[106]
	[Hbet]_2_[NTf_2_]Cl	[106]
CaO	[P_66614_]Cl/aq. HCl	[108]
	[Hbet][NTf_2_]	[106]
	[Hbet]_2_[NTf_2_]Cl	[106]
	[N_111_C_2_OSO_3_H][NTf_2_]/H_2_O	[109]
CdO	[Hbet][NTf_2_]/H_2_O and derivates	[102,104]
CoO	[P_66614_]Cl/aq. HCl	[108]
	[NRRRC_3_SO_3_H][NTf_2_]/H_2_O	[105]
	[PRRRC_3_SO_3_H][NTf_2_]/H_2_O	[105]
Co_3_O_4_	[Hbet][NTf_2_]	[106]
	[Hbet]_2_[NTf_2_]Cl	[106]
	[NRRRSO_3_H][NTf_2_]/H_2_O	[105]
	[PRRRSO_3_H][NTf_2_]/H_2_O	[105]
	[NRRH–SO_3_H][NTf_2_]/[emim]Cl	[110]
	[N_111_C_2_OSO_3_H][NTf_2_]/H_2_O	[109]
Cr_2_O_3_	[NRRRC_3_SO_3_H][NTf_2_]/H_2_O	[105]
	[PRRRC_3_SO_3_H][NTf_2_]/H_2_O	[105]
Cu_2_O	[Hbet][NTf_2_]	[106]
	[Hbet]_2_[NTf_2_]Cl	[106]
CuO	[P_66614_]Cl/aq. HCl	[108]
	[emim]Cl	[101]
	[emim][OAc]	[101]
	[Hbet][NTf_2_]/H_2_O and derivates	[102,104]
	[Hbet][NTf_2_]	[106]
	[Hbet]_2_[NTf_2_]Cl	[106]
	[NRRRC_3_SO_3_H][NTf_2_]/H_2_O	[105]
	[PRRRC_3_SO_3_H][NTf_2_]/H_2_O	[105]
	[NRRH–SO_3_H][NTf_2_]/[emim]Cl	[110]
	[N_111_C_2_OSO_3_H][NTf_2_]/H_2_O	[109]
Dy_2_O_3_	[Hbet][NTf_2_]/H_2_O and derivates	[102,104]
	[NRRRC_3_SO_3_H][NTf_2_]/H_2_O	[105]
	[PRRRC_3_SO_3_H][NTf_2_]/H_2_O	[105]
	[NRRH–SO_3_H][NTf_2_]/[emim]Cl	[110]
Er_2_O_3_	[Hbet][NTf_2_]/H_2_O and derivates	[102,104]
Eu_2_O_3_	[bmim][NTf_2_]/aq. HNO_3_	[111]
	[Hbet][NTf_2_]/H_2_O and derivates	[102,104]
Fe_2_O_3_	[P_66614_]Cl/aq. HCl	[108]
	[Hbet]_2_[NTf_2_]Cl	[106]
	[NRRRC_3_SO_3_H][NTf_2_]/H_2_O	[105]
	[PRRRC_3_SO_3_H][NTf_2_]/H_2_O	[105]
	[NRRH–SO_3_H][NTf_2_]/[emim]Cl	[110]
	[N_111_C_2_OSO_3_H][NTf_2_]/H_2_O	[109]
Gd_2_O_3_	[Hbet][NTf_2_]/H_2_O and derivates	[102,104]
HgO	[Hbet][NTf_2_]/H_2_O and derivates	[102,104]
Ho_2_O_3_	[Hbet][NTf_2_]/H_2_O and derivates	[102,104]
La_2_O_3_	[Hbet][NTf_2_]/H_2_O and derivates	[102,104]
	[NRRRC_3_SO_3_H][NTf_2_]/H_2_O	[105]
	[PRRRC_3_SO_3_H][NTf_2_]/H_2_O	[105]
	[NRRH–SO_3_H][NTf_2_]/[emim]Cl	[110]
	[N_111_C_2_OSO_3_H][NTf_2_]/H_2_O	[109]
Li_2_O	Ethylene carbonate/AlCl_3_	[112]
Lu_2_O_3_	[Hbet][NTf_2_]/H_2_O and derivates	[102,104]
MgO	[Hbet][NTf_2_]	[106]
	[Hbet]_2_[NTf_2_]Cl	[106]
MnO	[P_66614_]Cl/aq. HCl	[108]
	[Hbet][NTf_2_]/H_2_O	[102,103]
	[Hbet][NTf_2_]	[106]
	[Hbet]_2_[NTf_2_]Cl	[106]
	[NRRRC_3_SO_3_H][NTf_2_]/H_2_O	[105]
	[PRRRC_3_SO_3_H][NTf_2_]/H_2_O	[105]
	[NRRH–SO_3_H][NTf_2_]/[emim]Cl	[110]
MnO_2_	[Hbet]_2_[NTf_2_]Cl	[106]
	[NRRH–SO_3_H][NTf_2_]/[emim]Cl	[110]
MoO_3_	[Hbet][NTf_2_]	[106]
	[Hbet]_2_[NTf_2_]Cl	[106]
Nd_2_O_3_	[bmim][NTf_2_]/aq. HNO_3_	[111]
	[Hbet][NTf_2_]/H_2_O and derivates	[102,104]
	[NRRRC_3_SO_3_H][NTf_2_]/H_2_O	[105]
	[PRRRC_3_SO_3_H][NTf_2_]/H_2_O	[105]
	[NRRH–SO_3_H][NTf_2_]/[emim]Cl	[110]
	[N_111_C_2_OSO_3_H][NTf_2_]/H_2_O	[109]
NiO	[P_66614_]Cl/aq. HCl	[108]
	[emim]Cl	[101]
	[emim][OAc]	[101]
	[Hbet][NTf_2_]/H_2_O and derivates	[102,103,104]
	[Hbet]_2_[NTf_2_]Cl	[106]
	[NRRRC_3_SO_3_H][NTf_2_]/H_2_O	[105]
	[PRRRC_3_SO_3_H][NTf_2_]/H_2_O	[105]
	[NRRH–SO_3_H][NTf_2_]/[emim]Cl	[110]
	[N_111_C_2_OSO_3_H][NTf_2_]/H_2_O	[109]
PbO	[Hbet][NTf_2_]/H_2_O and derivates	[103,104,113]
	[Hbet][NTf_2_]	[106]
	[Hbet]_2_[NTf_2_]Cl	[106]
PbO_2_	[Hbet][NTf_2_]/H_2_O	[113]
	[Hbet][NTf_2_]	[106]
	[Hbet]_2_[NTf_2_]Cl	[106]
PdO	[Hbet][NTf_2_]/H_2_O and derivates	[102,104]
Pr_6_O_11_	[bmim][NTf_2_]/aq. HNO_3_	[111]
	[Hbet][NTf_2_]/H_2_O and derivates	[104,114]
PuO_2_	[Hbet][NTf_2_]/H_2_O	[18]
Sc_2_O_3_	Derivates of [Hbet][NTf_2_]/H_2_O	[104]
Sm_2_O_3_	[Hbet][NTf_2_]/H_2_O and derivates	[102,104]
SnO	[Hbet]_2_[NTf_2_]Cl	[106]
SrO	[Hbet][NTf_2_]	[106]
	[Hbet]_2_[NTf_2_]Cl	[106]
Tb_4_O_7_	[Hbet][NTf_2_]/H_2_O and derivates	[102,104]
ThO_2_	[Hbet]_2_[NTf_2_]Cl	[106]
TiO_2_	[NRRRC_3_SO_3_H][NTf_2_]/H_2_O	[105]
	[PRRRC_3_SO_3_H][NTf_2_]/H_2_O	[105]
Tm_2_O_3_	[Hbet][NTf_2_]/H_2_O and derivates	[102,104]
UO_2_	[emim]Cl/FeCl_3_	[115]
	[bmim]Cl/FeCl_3_	[115]
	[bdmim]Cl/FeCl_3_	[115]
	[bmim][NTf_2_]/aq. HNO_3_	[111]
	[emim][F(HF)n] (n = 2, 3)	[116]
	[Hbet][NTf_2_]/H_2_O	[117]
UO_3_	[emim]Cl/AlCl_3_	[118]
	[pdmim]Cl/AlCl_3_	[118]
	[bmim][NTf_2_]/aq. HNO_3_	[111]
	[dmah][NTf_2_]	[119]
	[emim][F(HF)n] (n = 2, 3)	[116]
	[Hbet][NTf_2_]/H_2_O and derivates	[102,104,117,120]
V_2_O_3_	[Hbet][NTf_2_]	[106]
	[Hbet]_2_[NTf_2_]Cl	[106]
V_2_O_5_	[emim]Cl/AlCl_3_	[121]
	[bmim]Cl/AlCl_3_	[121]
	[bpyr]Cl/AlCl_3_	[107]
	[Hbet][NTf_2_]	[106]
	[Hbet]_2_[NTf_2_]Cl	[106]
Y_2_O_3_	[Hbet][NTf_2_]/H_2_O and derivates	[102,104]
	[NRRRC_3_SO_3_H][NTf_2_]/H_2_O	[105]
	[PRRRC_3_SO_3_H][NTf_2_]/H_2_O	[105]
Yb_2_O_3_	[Hbet][NTf_2_]/H_2_O and derivates	[102,104]
WO_3_	[NRRRC_3_SO_3_H][NTf_2_]/H_2_O	[105]
	[PRRRC_3_SO_3_H][NTf_2_]/H_2_O	[105]
ZnO	[P_66614_]Cl/aq. HCl	[108]
	[emim]Cl	[101]
	[emim][OAc]	[101]
	[omim][OTf]	[122]
	[Hbet][NTf_2_]/H_2_O and derivates	[102,103,104]
	[Hbet][NTf_2_]	[106]
	[Hbet]_2_[NTf_2_]Cl	[106]
	[NRRRC_3_SO_3_H][NTf_2_]/H_2_O	[105]
	[PRRRC_3_SO_3_H][NTf_2_]/H_2_O	[105]
	[NRRH–SO_3_H][NTf_2_]/[emim]Cl	[110]

**Table 3:** Overview about metal oxides dissolved in different DESs as well as corresponding references.

Metal Oxide	Solvent	Reference
CoO	Choline chloride-malonic acid (1:1)	[133]
Co_3_O_4_	Choline chloride-malonic acid (1:1)	[133]
	Choline chloride-*p*-toluenesulfonic acid (1:2; 1:2; 2:1)	[134]
CrO_3_	Choline chloride-urea (1:2)	[133]
	Choline chloride-malonic acid (1:1)	[133]
Cu_2_O	Choline chloride-urea (1:2)	[133,135,136]
	Choline chloride-malonic acid (1:1)	[133]
	Choline chloride-ethylene glycol (1:2)	[133]
	Choline chloride-*p*-toluenesulfonic acid (1:2; 1:2; 2:1)	[134]
CuO	Choline chloride-urea (1:2)	[135,137]
	Choline chloride-malonic acid (1:1)	[133,138]
	Choline chloride-oxalic acid (1:1)	[138]
	Choline chloride-phenylpropionic acid (1:2)	[138]
	Choline chloride-*p*-toluenesulfonic acid (1:2; 1:2; 2:1)	[134]
Eu_2_O_3_	Ethylene glycol-maleic acid (1:1; 2:1; 4:1; 6:1)	[139]
	Ethylene glycol-citric acid (4:1)	[139]
	1,2-Propanediol-maleic acid (4:1)	[139]
	Glycerol-maleic acid (4:1)	[139]
	1,4-Butanediol-maleic acid (4:1)	[139]
FeO	Choline chloride-malonic acid (1:1)	[133]
Fe_2_O_3_	Choline chloride-malonic acid (1:1)	[133]
	Choline chloride-*p*-toluenesulfonic acid (1:2; 1:2; 2:1)	[134]
Fe_3_O_4_	Choline chloride-malonic acid (1:1)	[133,138]
	Choline chloride-oxalic acid (1:1)	[138]
	Choline chloride-phenylpropionic acid (1:2)	[138]
	Choline chloride-*p*-toluenesulfonic acid (1:2; 1:2; 2:1)	[134]
Gd_2_O_3_	Ethylene glycol-maleic acid (4:1)	[139]
In_2_O_3_	Choline chloride-*p*-toluenesulfonic acid (1:2; 1:2; 2:1)	[134]
La_2_O_3_	Ethylene glycol-maleic acid (1:1; 2:1; 4:1; 6:1)	[139]
	Ethylene glycol-citric acid (4:1)	[139]
	1,2-Propanediol-maleic acid (4:1)	[139]
	Glycerol-maleic acid (4:1)	[139]
MnO	Choline chloride-malonic acid (1:1)	[133]
	Choline chloride-*p*-toluenesulfonic acid (1:2; 1:2; 2:1)	[134]
Mn_2_O_3_	Choline chloride-malonic acid (1:1)	[133]
MnO_2_	Choline chloride-urea (1:2)	[135]
	Choline chloride-malonic acid (1:1)	[133]
	Choline chloride-*p*-toluenesulfonic acid (1:2; 1:2; 2:1)	[134]
MoO_3_	Choline chloride-urea (1:2)	[140]
Nd_2_O_3_	Ethylene glycol-maleic acid (4:1)	[139]
NiO	Choline chloride-urea (1:2)	[135]
	Choline chloride-malonic acid (1:1)	[133]
Ni_2_O_3_	Choline chloride-urea (1:2)	[141]
PbO	Choline chloride-urea (1:2)	[142,143,144]
PbO_2_	Choline chloride-urea (1:2)	[135]
	Choline chloride-*p*-toluenesulfonic acid (1:2; 1:2; 2:1)	[134]
Pr_6_O_11_	Ethylene glycol-maleic acid (4:1)	[139]
Sm_2_O_3_	Ethylene glycol-maleic acid (4:1)	[139]
V_2_O_3_	Choline chloride-urea (1:2)	[133]
	Choline chloride-malonic acid (1:1)	[133]
	Choline chloride-ethylene glycol (1:2)	[133]
V_2_O_5_	Choline chloride-urea (1:2)	[133,145]
	Choline chloride-malonic acid (1:1)	[133]
	Choline chloride-ethylene glycol (1:2)	[133]
ZnO	Choline chloride-urea (1:2)	[133,135,137,146,147]
	Choline chloride-malonic acid (1:1)	[133,138]
	Choline chloride-oxalic acid (1:1)	[138]
	Choline chloride-phenylpropionic acid (1:2)	[138]
	Choline chloride-ethylene glycol (1:2)	[133]
	Choline chloride-*p*-toluenesulfonic acid (1:2; 1:2; 2:1)	[134]
	[bmim]Cl-urea (1:1; 1:2)	[148,149]
	[emim]Cl-urea (1:1; 1:2)	[149,150]
	[amim]Cl-urea (1:1)	[149]
